# (2*E*)-1-(3-Chloro­phen­yl)-3-phenyl­prop-2-en-1-one

**DOI:** 10.1107/S1600536809053458

**Published:** 2009-12-16

**Authors:** Jerry P. Jasinski, Ray J. Butcher, B. Narayana, K. Veena, H. S. Yathirajan

**Affiliations:** a Department of Chemistry, Keene State College, 229 Main Street, Keene, NH 03435-2001, USA; b Department of Chemistry, Howard University, 525 College Street NW, Washington, DC 20059, USA; c Department of Studies in Chemistry, Mangalore University, Manalaganotri 574 199, India; d Department of Studies in Chemistry, University of Mysore, Manasagangotri, Mysore 570 006, India

## Abstract

In the title compound, C_15_H_11_ClO, the dihedral angle between the mean planes of the benzene ring and the chloro-substituted benzene ring is 48.8 (3)°. The dihedral angles between the mean plane of the prop-2-ene-1-one group and the mean planes of the 3-chloro­phenyl and benzene rings are 27.0 (4) and 27.9 (3)°, respectively. In the crystal, weak inter­molecular C—H⋯π-ring inter­actions occur.

## Related literature

For background to chalcones, see: Chen *et al.* (1994 ▶); Marais *et al.* (2005 ▶); Poornesh *et al.* (2009 ▶); Ram *et al.* (2000 ▶); Sarojini *et al.* (2006 ▶); Shettigar *et al.* (2006 ▶, 2008 ▶); Troeberg *et al.* (2000 ▶). For related structures, see: Jasinski *et al.* (2007 ▶); Li & Su (1994 ▶).
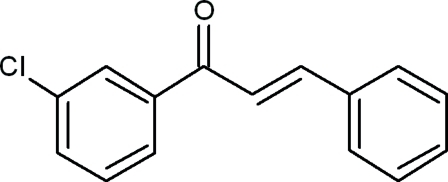


## Experimental

### 

#### Crystal data

C_15_H_11_ClO
*M* *_r_* = 242.69
Triclinic, 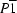

*a* = 5.8388 (7) Å
*b* = 7.5975 (11) Å
*c* = 13.1300 (16) Å
α = 83.182 (11)°
β = 89.422 (10)°
γ = 86.662 (11)°
*V* = 577.35 (13) Å^3^
*Z* = 2
Cu *K*α radiation
μ = 2.74 mm^−1^
*T* = 110 K
0.50 × 0.32 × 0.28 mm

#### Data collection

Oxford Diffraction Xcalibur diffractometer with a Ruby (Gemini Cu) detector
Absorption correction: multi-scan (*CrysAlis RED*; Oxford Diffraction, 2007 ▶) *T*_min_ = 0.541, *T*_max_ = 1.000
3661 measured reflections
2243 independent reflections
2148 reflections with *I* > 2σ(*I*)
*R*_int_ = 0.017

#### Refinement

*R*[*F* ^2^ > 2σ(*F* ^2^)] = 0.036
*wR*(*F* ^2^) = 0.099
*S* = 1.02
2243 reflections
154 parameters
H-atom parameters constrained
Δρ_max_ = 0.34 e Å^−3^
Δρ_min_ = −0.22 e Å^−3^

### 

Data collection: *CrysAlis PRO* (Oxford Diffraction, 2007 ▶); cell refinement: *CrysAlis RED* (Oxford Diffraction, 2007 ▶); data reduction: *CrysAlis RED*; program(s) used to solve structure: *SHELXS97* (Sheldrick, 2008 ▶); program(s) used to refine structure: *SHELXL97*) (Sheldrick, 2008 ▶); molecular graphics: *SHELXTL* (Sheldrick, 2008 ▶); software used to prepare material for publication: *SHELXTL*.

## Supplementary Material

Crystal structure: contains datablocks global, I. DOI: 10.1107/S1600536809053458/tk2596sup1.cif

Structure factors: contains datablocks I. DOI: 10.1107/S1600536809053458/tk2596Isup2.hkl

Additional supplementary materials:  crystallographic information; 3D view; checkCIF report

## Figures and Tables

**Table 1:** Hydrogen-bond geometry (Å, °)

*D*—H⋯*A*	*D*—H	H⋯*A*	*D*⋯*A*	*D*—H⋯*A*
C2—H2*A*⋯*Cg*2^i^	0.95	2.90	3.5541 (16)	127
C5—H5*A*⋯*Cg*2^ii^	0.95	2.90	3.5338 (17)	125
C12—H12*A*⋯*Cg*1^iii^	0.95	2.92	3.6040 (17)	130

Symmetry codes: (i) 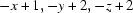; (ii) 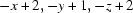; (iii) 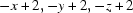. *Cg*1 is the centroid of the C1–C6 ring and *Cg*2 is the centroid of the C10–C15 ring.

**Fig. 1.**
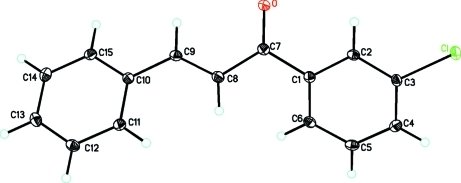
Molecular structure of (I), showing the atom labeling scheme and 50% probability displacement ellipsoids.

## supplementary crystallographic information

### Comment

Chalcones are known as the precursors of all flavonoid type natural products in biosynthesis (Marais *et al.*, 2005). Chalcones exhibit various biological activities like insecticidal, antimicrobial, antichinoviral, antipicorniviral and bacteriostatic properties. Azachalcones, the derivatives of chalcones with an annular nitrogen atom in the phenyl ring, were reported to have a wide range of biological activities, such as antibacterial, antituberculostatic and anti-inflammatory. An important feature of chalcones are their ability to act as activated unsaturated systems in conjugated addition of carbanions in presence of suitable basic catalysts. Many chalcones have been described for their high antimalarial activity, probably as a result of Michael addition of nucleophilic species to the double bond of the enone (Troeberg *et al.*, 2000; Ram *et al.*, 2000). Licochalcone A, isolated from Chinese liquorice roots, has been reported as being highly effective in chloroquine resistant Plasmodium falciparum strains in a [3H] hypoxanthine uptake assay (Chen *et al.*, 1994). Chalcones are also finding applications as organic non-linear optical materials (NLO) due to their good SHG conversion efficiencies (Sarojini *et al.*, 2006). Recently, non-linear optical studies on a few chalcones and their derivatives were reported (Poornesh *et al.*, 2009; Shettigar *et al.*, 2006; 2008). In continuation with our studies of chalcones (Jasinski *et al.*, 2007) and their derivatives and owing to the importance of these flavanoid analogs, the title chalcone, (I), was synthesized and its crystal structure reported herein.

The title compound, (I), is a chalcone with 3-chlorophenyl and benzene rings bonded at the opposite ends of a propenone group, the biologically active region (Fig.1). The dihedral angle between mean planes of the benzene and chloro substituted benzene rings is 48.8 (3)° as compared to 14.3 (7)° in the 4-chloro benzene analogue compound (Li & Su, 1994). The angles between the mean plane of the prop-2-ene-1-one group and the mean planes of the 3-chlorophenyl and benzene rings are 27.0 (4)° and 27.9 (3)°, respectively, as compared to 19.4 (2)° and 11.9 (9)° in the aforementioned 4-chloro benzene compound. While no classical hydrogen bonds are present, weak intermolecular C–H···π-ring interactions are observed which contribute to the stability of crystal packing (Table 1).

### Experimental

50% KOH was added to a mixture of 3-chloro acetophenone (0.01 mol) and benzaldehyde (0.01 mol) in 25 ml of ethanol (Scheme 2). The mixture was stirred for an hour at room temperature and the precipitate was collected by filtration and purified by recrystallization from ethanol. The single-crystal was grown from ethyl acetate by slow evaporation method and yield of the compound was 72% (m.p.: 354–356 K). Analytical data for C_15_H_11_ClO: Found (Calculated): C%: 74.19 (74.23); H%: 4.55 (4.57).

### Refinement

All of the H atoms were placed in their calculated positions and then refined using the riding model with C—H = 0.95 Å, and with *U*_iso_(H) = 1.17–1.22*U*_eq_(C).

### Crystal data

**Table:**

C_15_H_11_ClO	*Z* = 2
*M**_r_* = 242.69	*F*(000) = 252
Triclinic, *P*1	*D*_x_ = 1.396 Mg m^−^^3^
Hall symbol: -P 1	Cu *K*α radiation, λ = 1.54184 Å
*a* = 5.8388 (7) Å	Cell parameters from 3077 reflections
*b* = 7.5975 (11) Å	θ = 5.9–73.8°
*c* = 13.1300 (16) Å	µ = 2.74 mm^−^^1^
α = 83.182 (11)°	*T* = 110 K
β = 89.422 (10)°	Prism, colorless
γ = 86.662 (11)°	0.50 × 0.32 × 0.28 mm
*V* = 577.35 (13) Å^3^	

### Data collection

**Table:**

Oxford Diffraction Xcalibur diffractometer with a Ruby (Gemini Cu) detector	2243 independent reflections
Radiation source: Enhance (Cu) X-ray Source	2148 reflections with *I* > 2σ(*I*)
graphite	*R*_int_ = 0.017
Detector resolution: 10.5081 pixels mm^-1^	θ_max_ = 73.8°, θ_min_ = 5.9°
ω scans	*h* = −7→5
Absorption correction: multi-scan (*CrysAlis RED*; Oxford Diffraction, 2007)	*k* = −9→9
*T*_min_ = 0.541, *T*_max_ = 1.000	*l* = −16→15
3661 measured reflections	

### Refinement

**Table:**

Refinement on *F*^2^	Primary atom site location: structure-invariant direct methods
Least-squares matrix: full	Secondary atom site location: difference Fourier map
*R*[*F*^2^ > 2σ(*F*^2^)] = 0.036	Hydrogen site location: inferred from neighbouring sites
*wR*(*F*^2^) = 0.099	H-atom parameters constrained
*S* = 1.02	*w* = 1/[σ^2^(*F*_o_^2^) + (0.0647*P*)^2^ + 0.2987*P*] where *P* = (*F*_o_^2^ + 2*F*_c_^2^)/3
2243 reflections	(Δ/σ)_max_ < 0.001
154 parameters	Δρ_max_ = 0.34 e Å^−^^3^
0 restraints	Δρ_min_ = −0.22 e Å^−^^3^

### Special details

**Table:**

Geometry. All e.s.d.'s (except the e.s.d. in the dihedral angle between two l.s. planes) are estimated using the full covariance matrix. The cell e.s.d.'s are taken into account individually in the estimation of e.s.d.'s in distances, angles and torsion angles; correlations between e.s.d.'s in cell parameters are only used when they are defined by crystal symmetry. An approximate (isotropic) treatment of cell e.s.d.'s is used for estimating e.s.d.'s involving l.s. planes.
Refinement. Refinement of *F*^2^ against ALL reflections. The weighted *R*-factor *wR* and goodness of fit *S* are based on *F*^2^, conventional *R*-factors *R* are based on *F*, with *F* set to zero for negative *F*^2^. The threshold expression of *F*^2^ > σ(*F*^2^) is used only for calculating *R*-factors(gt) *etc*. and is not relevant to the choice of reflections for refinement. *R*-factors based on *F*^2^ are statistically about twice as large as those based on *F*, and *R*- factors based on ALL data will be even larger.

### Fractional atomic coordinates and isotropic or equivalent isotropic displacement parameters (Å^2^)

**Table:**

	*x*	*y*	*z*	*U*_iso_*/*U*_eq_	
Cl	0.32652 (6)	0.68985 (5)	0.56518 (3)	0.02296 (15)	
O	0.27901 (18)	0.72419 (15)	0.96930 (8)	0.0224 (3)	
C1	0.5736 (2)	0.67070 (18)	0.84979 (11)	0.0160 (3)	
C2	0.4270 (2)	0.69988 (18)	0.76536 (11)	0.0162 (3)	
H2A	0.2759	0.7510	0.7719	0.019*	
C3	0.5060 (3)	0.65289 (19)	0.67198 (11)	0.0165 (3)	
C4	0.7258 (3)	0.57602 (19)	0.66031 (12)	0.0189 (3)	
H4A	0.7771	0.5448	0.5956	0.023*	
C5	0.8677 (3)	0.54612 (19)	0.74507 (12)	0.0193 (3)	
H5A	1.0170	0.4916	0.7387	0.023*	
C6	0.7950 (2)	0.59463 (19)	0.83940 (12)	0.0177 (3)	
H6A	0.8955	0.5762	0.8966	0.021*	
C7	0.4848 (3)	0.72066 (19)	0.95085 (11)	0.0175 (3)	
C8	0.6548 (3)	0.7663 (2)	1.02521 (12)	0.0192 (3)	
H8A	0.8081	0.7850	1.0035	0.023*	
C9	0.5952 (2)	0.78129 (19)	1.12239 (11)	0.0170 (3)	
H9A	0.4423	0.7560	1.1418	0.020*	
C10	0.7434 (2)	0.83314 (19)	1.20198 (11)	0.0161 (3)	
C11	0.9534 (3)	0.90987 (19)	1.17865 (11)	0.0180 (3)	
H11A	1.0036	0.9293	1.1094	0.022*	
C12	1.0875 (3)	0.95730 (19)	1.25607 (12)	0.0194 (3)	
H12A	1.2287	1.0103	1.2396	0.023*	
C13	1.0172 (3)	0.9280 (2)	1.35792 (12)	0.0217 (3)	
H13A	1.1107	0.9600	1.4108	0.026*	
C14	0.8103 (3)	0.8519 (2)	1.38218 (12)	0.0221 (3)	
H14A	0.7623	0.8313	1.4517	0.026*	
C15	0.6734 (3)	0.80576 (19)	1.30478 (12)	0.0185 (3)	
H15A	0.5309	0.7552	1.3217	0.022*	

### Atomic displacement parameters (Å^2^)

**Table:**

	*U*^11^	*U*^22^	*U*^33^	*U*^12^	*U*^13^	*U*^23^
Cl	0.0222 (2)	0.0308 (2)	0.0164 (2)	0.00195 (15)	−0.00390 (14)	−0.00610 (14)
O	0.0149 (5)	0.0340 (6)	0.0190 (5)	−0.0032 (4)	0.0000 (4)	−0.0044 (4)
C1	0.0171 (7)	0.0139 (6)	0.0173 (7)	−0.0054 (5)	0.0001 (5)	−0.0010 (5)
C2	0.0140 (7)	0.0149 (7)	0.0198 (7)	−0.0028 (5)	0.0000 (5)	−0.0019 (5)
C3	0.0177 (7)	0.0173 (7)	0.0152 (7)	−0.0036 (5)	−0.0023 (5)	−0.0031 (5)
C4	0.0209 (7)	0.0165 (7)	0.0203 (7)	−0.0034 (5)	0.0034 (6)	−0.0050 (6)
C5	0.0145 (7)	0.0156 (7)	0.0277 (8)	−0.0003 (5)	0.0009 (6)	−0.0030 (6)
C6	0.0161 (7)	0.0160 (7)	0.0208 (7)	−0.0032 (5)	−0.0023 (5)	−0.0001 (5)
C7	0.0175 (7)	0.0168 (7)	0.0179 (7)	−0.0030 (5)	−0.0005 (5)	−0.0002 (5)
C8	0.0166 (7)	0.0213 (7)	0.0199 (7)	−0.0033 (6)	−0.0009 (6)	−0.0023 (6)
C9	0.0143 (7)	0.0154 (7)	0.0212 (7)	−0.0003 (5)	−0.0004 (5)	−0.0021 (5)
C10	0.0155 (7)	0.0139 (6)	0.0188 (7)	0.0015 (5)	−0.0010 (5)	−0.0030 (5)
C11	0.0173 (7)	0.0175 (7)	0.0189 (7)	−0.0001 (5)	0.0011 (5)	−0.0024 (5)
C12	0.0161 (7)	0.0156 (7)	0.0264 (8)	−0.0011 (5)	−0.0017 (6)	−0.0020 (6)
C13	0.0228 (8)	0.0201 (7)	0.0227 (8)	0.0003 (6)	−0.0064 (6)	−0.0046 (6)
C14	0.0268 (8)	0.0221 (8)	0.0171 (7)	0.0001 (6)	0.0010 (6)	−0.0027 (6)
C15	0.0172 (7)	0.0166 (7)	0.0220 (8)	−0.0010 (5)	0.0029 (6)	−0.0032 (6)

### Geometric parameters (Å, °)

**Table:**

Cl—C3	1.7452 (15)	C8—H8A	0.9500
O—C7	1.2226 (19)	C9—C10	1.467 (2)
C1—C2	1.396 (2)	C9—H9A	0.9500
C1—C6	1.397 (2)	C10—C15	1.402 (2)
C1—C7	1.502 (2)	C10—C11	1.404 (2)
C2—C3	1.385 (2)	C11—C12	1.383 (2)
C2—H2A	0.9500	C11—H11A	0.9500
C3—C4	1.393 (2)	C12—C13	1.391 (2)
C4—C5	1.383 (2)	C12—H12A	0.9500
C4—H4A	0.9500	C13—C14	1.387 (2)
C5—C6	1.389 (2)	C13—H13A	0.9500
C5—H5A	0.9500	C14—C15	1.389 (2)
C6—H6A	0.9500	C14—H14A	0.9500
C7—C8	1.483 (2)	C15—H15A	0.9500
C8—C9	1.335 (2)		
			
C2—C1—C6	120.12 (14)	C7—C8—H8A	119.6
C2—C1—C7	118.15 (13)	C8—C9—C10	126.15 (14)
C6—C1—C7	121.73 (13)	C8—C9—H9A	116.9
C3—C2—C1	118.72 (13)	C10—C9—H9A	116.9
C3—C2—H2A	120.6	C15—C10—C11	118.75 (13)
C1—C2—H2A	120.6	C15—C10—C9	119.08 (13)
C2—C3—C4	121.96 (13)	C11—C10—C9	122.18 (13)
C2—C3—Cl	119.50 (11)	C12—C11—C10	120.27 (14)
C4—C3—Cl	118.54 (11)	C12—C11—H11A	119.9
C5—C4—C3	118.53 (14)	C10—C11—H11A	119.9
C5—C4—H4A	120.7	C11—C12—C13	120.46 (14)
C3—C4—H4A	120.7	C11—C12—H12A	119.8
C4—C5—C6	120.91 (14)	C13—C12—H12A	119.8
C4—C5—H5A	119.5	C14—C13—C12	119.92 (14)
C6—C5—H5A	119.5	C14—C13—H13A	120.0
C5—C6—C1	119.74 (14)	C12—C13—H13A	120.0
C5—C6—H6A	120.1	C13—C14—C15	119.97 (14)
C1—C6—H6A	120.1	C13—C14—H14A	120.0
O—C7—C8	122.19 (14)	C15—C14—H14A	120.0
O—C7—C1	120.25 (13)	C14—C15—C10	120.63 (14)
C8—C7—C1	117.56 (13)	C14—C15—H15A	119.7
C9—C8—C7	120.80 (14)	C10—C15—H15A	119.7
C9—C8—H8A	119.6		
			
C6—C1—C2—C3	−0.3 (2)	O—C7—C8—C9	12.5 (2)
C7—C1—C2—C3	−179.41 (12)	C1—C7—C8—C9	−168.11 (14)
C1—C2—C3—C4	0.7 (2)	C7—C8—C9—C10	−177.12 (13)
C1—C2—C3—Cl	−179.48 (10)	C8—C9—C10—C15	−166.17 (15)
C2—C3—C4—C5	0.1 (2)	C8—C9—C10—C11	14.0 (2)
Cl—C3—C4—C5	−179.76 (11)	C15—C10—C11—C12	0.0 (2)
C3—C4—C5—C6	−1.2 (2)	C9—C10—C11—C12	179.79 (13)
C4—C5—C6—C1	1.6 (2)	C10—C11—C12—C13	0.6 (2)
C2—C1—C6—C5	−0.8 (2)	C11—C12—C13—C14	−0.5 (2)
C7—C1—C6—C5	178.26 (12)	C12—C13—C14—C15	−0.2 (2)
C2—C1—C7—O	26.0 (2)	C13—C14—C15—C10	0.9 (2)
C6—C1—C7—O	−153.08 (14)	C11—C10—C15—C14	−0.7 (2)
C2—C1—C7—C8	−153.42 (13)	C9—C10—C15—C14	179.46 (13)
C6—C1—C7—C8	27.50 (19)		

### Hydrogen-bond geometry (Å, °)

**Table:**

Cg1 is the centroid of the C1–C6 ring and Cg2 is the centroid of the C10–C15 ring.

**Table:**

*D*—H···*A*	*D*—H	H···*A*	*D*···*A*	*D*—H···*A*
C2—H2A···Cg2^i^	0.95	2.90	3.5541 (16)	127
C5—H5A···Cg2^ii^	0.95	2.90	3.5338 (17)	125
C12—H12A···Cg1^iii^	0.95	2.92	3.6040 (17)	130

Symmetry codes: (i) −*x*+1, −*y*+2, −*z*+2; (ii) −*x*+2, −*y*+1, −*z*+2; (iii) −*x*+2, −*y*+2, −*z*+2.
